# Lack of an association between dietary patterns and adiposity among primary school children in Kilimanjaro Tanzania

**DOI:** 10.1186/s40795-022-00529-4

**Published:** 2022-04-21

**Authors:** Mary Vincent Mosha, Heavenlight A. Paulo, Sia E. Msuya, Heiner Grosskurth, Suzanne Filteau

**Affiliations:** 1 Institute of Public Health, Kilimanjaro Christian Medical University College (KCMUCO), Moshi, Tanzania; 2 Department of Epidemiology and Biostatistics, Muhimbili University of Health and Allied Sciences, Dar es Salaam, Tanzania; 3 London School of Hygiene and Tropical Medicine (LSHTM), London, UK; 4 Mwanza Intervention Trials Unit (MITU), National Institute for Medical Research, Mwanza, Tanzania

**Keywords:** Dietary patterns, Food frequency questionnaire, School children, Adiposity, Factor analysis, Tanzania

## Abstract

**Introduction:**

Healthy dietary habits prevent childhood overweight and obesity and the risk of non-communicable diseases (NCDs) later in life. We examined dietary patterns and their association with adiposity among primary school children in northern Tanzania.

**Methods:**

Dietary data was collected by 24-h recall and food frequency questionnaire (FFQ) for 1170 primary school children aged 9 – 11 years from 20 primary schools in the Kilimanjaro region. Factor analysis and FFQ data were used to identify dietary patterns. Children were categorized into terciles of their adherence to each dietary pattern. Multilevel logistic regression was used to evaluate the association of dietary pattern terciles with adiposity indicators: body mass index z–scores (BMI z scores), body fat percentage by bioelectrical impedance, triceps, subscapular skinfold thicknesses, and waist circumference.

**Results:**

Fifteen percent of children had BMI Z > 1.0, indicating overweight or obesity. Two dietary patterns were identified by factor analysis: a healthy pattern characterized by frequent consumption of fruits and vegetables; and a mixed dietary pattern characterized by intake of fatty snacks, sweets and sugar snacks, sugary beverages, meat and alternatives, milk, and milk products. After adjusting for potential confounders, for both models: model 1 (age and sex), and model 2 (age, sex, school type, time spent walking to school, district [urban/ rural], availability of television and electronic gadgets at home and neighbourhood playground); we found no significant associations between dietary patterns and adiposity measures.

**Conclusion:**

Dietary patterns were not associated with adiposity in Tanzanian primary school children, possibly because of limitations of the FFQ, which did not record information on portion sizes. Future research should focus on understanding the key foods / snacks consumed by school children, portion sizes and their long-term effects on adiposity in children.

**Supplementary Information:**

The online version contains supplementary material available at 10.1186/s40795-022-00529-4.

## Introduction

Globally, over 340 million children and adolescents aged 5—19 were either overweight or obese in 2016 [1]. Childhood overweight and obesity have increased in low- and middle-income countries (LMICs) where under-nutrition persists. In Africa, the prevalence of overweight and obesity was 12.7% in 2020 [2]. A recent review in Sub Saharan Africa reported the rapid increase in childhood overweight and obesity [3]. In Tanzania, studies conducted between 2010 to 2021 reported a prevalence ranging from 5.6% to 18.0% [4–7].

Diet is an important modifiable factor for preventing overweight and obesity [8]. A healthy diet may prevent excess weight gain, excess body fat, and associated risks of premature death and disability from nutrition-related, non-communicable diseases, including cardiovascular diseases and diabetes [9, 10]. Obese children tend to remain obese adults, which increases the risk for many co-morbidities [11]. Many LMICs are experiencing a nutrition transition due to globalization and urbanization. Availability of cheaper imported foods primarily in urban areas encourages families to change their dietary patterns from traditional boiled staple / local foods (e.g., cassava, plantain, and potatoes) to energy-dense, deep-fried, refined foods, sugary snacks and beverages, and low consumption of fruits and vegetables [12, 13]. Children are surrounded by a complex food environment that promotes unhealthy foods that are energy-dense and lacking in nutrients. Parental preferences on food choices and other environmental factors might promote children’s unhealthy or healthy eating habits [14, 15]. In Tanzania, school children often access deep-fried snacks and processed foods, (e.g., fried cassava and potatoes, “mandazi” (Tanzanian doughnuts), and flavoured ice pops from street vendors) [16].

The relationship between overweight / obesity, adiposity and dietary behaviours in children has been established elsewhere [17–20]. One review in high-income countries showed that unhealthy dietary patterns might be associated with adiposity in children at different ages; the review concluded by encouraging decreased intake of high energy-dense foods and increased consumption of fruits and vegetables [20, 21].

There is limited information about dietary patterns and adiposity for children in LMICs. In Tanzania, few studies reported the prevalence while information on the dietary patterns and adiposity was insufficient. In this study, we modified, using data from 24-h recall, a standardized food frequency questionnaire (FFQ) which was developed and successfully used in research on the International Study on Childhood Obesity, Lifestyle, and Environment (ISCOLE), to examine the association between diet, and adiposity [22]. We examined dietary patterns among Tanzanian primary school children and their association with different measures of adiposity.

## Methods

### Study setting

From August to November 2019, we conducted a school-based cross-sectional study in 20 primary schools in the Kilimanjaro Region, Northern Tanzania. Kilimanjaro is one of the 31 administrative regions in Tanzania and comprises seven administrative districts. Two districts, Moshi urban and Moshi rural, were purposely selected to represent urban and rural settings. Moshi urban has 48 primary schools (35 government schools and 13 private schools) and 33,207 students. Moshi rural has 272 primary schools (252 government: 20 private) and 81,297 students.

### Study population

A multistage sampling technique was used to select the study participants. We randomly selected eight wards from each of the two districts. Primary schools from the selected wards were stratified by type of schools (government or private). A simple random sampling was used to select 20 primary schools (10 from each district). Enrolment in government or private schools was regarded as a proxy indicator for lower or higher socio-economic status. We excluded children from boarding schools as they usually follow the same school menu. A list of all students aged 9 to 11 years from classes 4 to 6 was obtained from the school attendance registers. Study participants were randomly selected from each school according to probability proportional to the size of schools from the list. This age band was set based on the assumption that children aged nine or older can express themselves and fill in questionnaires with guidance. Nevertheless, we excluded 24 children who could not understand the questions and express themselves.

### Sample size estimation

Sample size estimations were based on the study’s primary aim: to estimate the prevalence of overweight and obesity [4]. A precision of ± 2.5% and a design effect of 1.3 was assumed since we recruited participants from clusters (schools). The final sample size was 1170 primary school children.

### Dietary intake assessment

#### Pilot study: 24-h dietary recall

We first conducted a pilot study to explore the food items usually consumed by children, using a 24-h dietary recall among 51 children (9 -11 years) randomly selected from four primary schools, two from each district (urban/ rural). A trained nutritionist asked children to recall their food and beverage intake in the previous 24 h. We followed the four-stage multipass technique to obtain information on detailed food items consumed in the past 24 h [23]. Information on snacking habits, especially during recess time at school, lunchtime, on the way back home, and at friends’ houses, were used to get specific details for sugary drinks, snacks, and high-calorie low nutrition snacks. Food models were used to aid in portion size estimations [23]. We used the Tanzania food composition table (TFCT) [24] to assign nutrient values and estimate different foods' energy and nutrient intake. For industrially manufactured foods mentioned by children that were not on the TFCT (e.g., Pringles [dehydrated processed potato crisps], and sausages) we obtained the information from food labels where possible. Nutritional information for each food was computed per 100 g and then converted to the consumed amount based on self-report. For children who could not estimate their portion size, the standardized portion sizes from TFCT were used to estimate their intake.

#### Food frequency questionnaire

For the main study of dietary intake, findings from the 24-h recall were used to adapt and modify the ISCOLE FFQ [22]. This standard FFQ has been used elsewhere to explore the association between diet and obesity [25–27]. We removed some food items which did not apply to Tanzanian children (e.g., low-fat milk, cheese, energy drinks: “Redbull, guru”, sports drinks “Powerade”) and added local foods / snacks reported frequently by children, (e.g., samosas, chips, fried cassava, fried plantain, flavoured ice pops (commonly made of tamarind, sugar and food colour), sweetened squeezed fruit juice, mandazi, milk and milk products (whole cow’s milk and cultured milk)). Children were asked about their usual intake of different foods in a typical week. Scores were generated from the FFQ responses to obtain servings per day as follows: never = 0 servings / day; once per day = 1 serving per day; once or twice per week = 0.21 servings per day; 3—6 times per week = 0.64 servings per day; and 2—3 times a day / every day more than once = 2.5 servings per day [28]. Trained research assistants guided children step by step on filling the FFQ to ensure they understood each food item, contents and answered them independently.

#### Defining food groups and subgroups

We used the information on local foods and snacks reported by children from the 24-h dietary recall to estimate the contribution of different foods to total fat, carbohydrates, and protein in the diet. Further, to create food categories, we aggregated the 15 food items from the FFQ into nine foods / food groups based on their nutrient composition and culinary uses (percentage contribution in the children’s diet). For example, we grouped intake of chocolates, cakes, sweets, biscuits and doughnuts as sweets and sugars. The food groups / subgroups were used for identifying dietary patterns.

### Study outcomes

Our study outcomes were five adiposity measures: BMI z–scores, waist circumference, body fat percentage by bioelectrical impedance, triceps, subscapular skinfolds; and associations between dietary patterns and measurements of adiposity.

### Adiposity measures

Anthropometric measurements were performed in duplicate following the National Health and Nutrition Examination Survey (NHANES) standard procedures [29], using calibrated equipment. Before taking any measures, we asked children to remove shoes, socks, hair ornaments, items from pockets, jewellery, and clothing other than their regular school uniform. Two trained research assistants took independent anthropometry and adiposity measurements from each child, and the average between the two measurements was calculated. Routinely, we checked for standard operating procedures and assessed the technical error of measurement (TEM) [30] to estimate the inter-observer measurements errors.

Height was measured to the nearest 0.1 cm using the TANITA height rod with children standing straight with the head in the Frankfurt plane. Weight and total body fat percentage were measured using bioelectric impedance analysis technology (BIA), TANITA model DC 430 MA. The BIA machine was set to deduct 0.5 kg (clothing weight) before measurements.

Waist circumference was measured to the nearest 0.1 cm using a non-elastic circumference tape. Children were asked to stand straight with the abdomen relaxed, arms at the sides, feet together and the measurement was taken from the narrowest girth. For obese children in whom we couldn’t identify the measurement point, we asked them to bend to the side, and the measure was taken from the point where the trunk folds.

Triceps and subscapular skinfolds were measured by Harpenden precision thickness calliper to the nearest 0.1 mm, on the right arm [23]. Triceps skinfold was measured at the midpoint of the upper right arm’s back. Subscapular skinfold was measured below and laterally to the shoulder blade’s angle, with the shoulder and arm relaxed.

### Statistical analysis

Analysis was performed by STATA version 15.1 (StataCorp, College Station, TX, USA). Probability plots were used to assess the distribution of variables and to check for normality. Continuous variables were summarized using means and standard deviations for data which are normally distributed, and medians and interquartile ranges for skewed distributed data. For categorical variables, frequencies and proportions were reported. WHO Anthroplus (STATA SE) was used to determine the BMI z-scores based on age and sex; thinness < -2 SD, normal weight between -2SD and 1SD, overweight >  + 1 and <  + 2SD, and obese ≥  + 2SD. We considered schools as clusters since data were collected from different school settings.

**Fig. 1:**
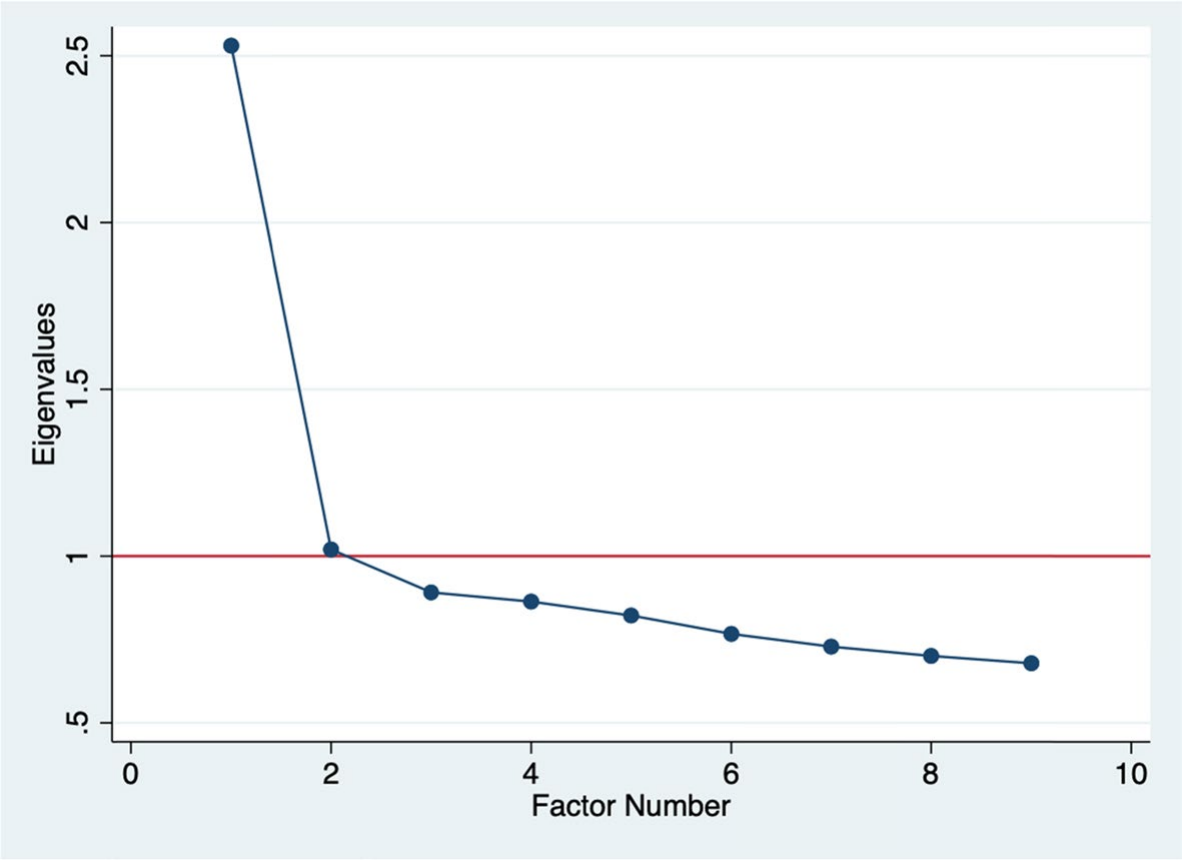
Scree plot for identification of dietary patterns by factor analysis

Based on the scores generated from the FFQ, we performed factor analysis, a data-driven method to derive the dietary patterns. The factor was loaded and rotated using varimax rotation to simplify the interpretation of the factors. The correlation between foods / food groups was examined using a Kaiser Meyer Olkin (KMO) test = 0.83, indicating that the correlation among the variables was sufficiently strong for factor analysis. We used a scree plot to examine the distribution of factors and to select the factors to retain. We retained factors with the eigenvalues greater than 1; factor 1 = eigenvalue 2.53 and factor 2 = eigenvalue 1.01 [Fig. 1].

Variables (food items / groups) with a factor loading of absolute ≥ 0.3 [31], were retained and used for labelling dietary patterns. When food items loaded higher than 0.3 in more than one factor, the factor with the highest loading was considered for factor labelling. Children were categorized into terciles of low, medium, or high adherence to each dietary pattern. Further, Chi-square test, analysis of variance (ANOVA), and Kruskal Wallis test were performed to explore participants characteristics across terciles of dietary patterns.

Variables potentially associated with dietary patterns, were selected for adjustment based on a recent study on overweight/obesity conducted in Tanzanian primary schools [4]. In model 1, we adjusted for age and sex. Model 2 adjusted for lifestyle factors: school type (private or government), time spent walking to school, district (rural or urban), availability of television and electronic gadgets at home, and neighbourhood playground.

Next, we examined the association between the identified dietary pattern terciles and different adiposity measures using multilevel linear regression. The dependent variables were logarithmically transformed before linear regression analysis because of the non-normal distributions. All analyses were two-tailed, and the significance level was set at 5%.

## Results

Table 1 describes the characteristics of study participants, anthropometry, and adiposity measures. About half of the children were girls, and the median BMI z-score was -0.58 (25^th^ and 75^th^ percentiles: -1.3, 0.3). The prevalence of overweight, obesity and thinness were 9.0%, 6.0%, and 10.3%, respectively.

**Table 1:** Characteristics of primary school children (*N* = 1170)

Variable	n (%)
**Age (years)**	
9	349 (29.8)
10	397 (33.9)
11	424 (36.2)
**Sex**	
Female	636 (54.3)
**Area of residence**	
Moshi Rural	601 (51.4)
Moshi Urban	569 (48.6)
**School ownership**	
Government	589 (50.3)
Private	581 (49.7)
**Nutritional status**	
BMI z-score > 1 (overweight/obese)	176 (15.0)
BMI z-score < -2 (thinness)	121 (10.3)
**Anthropometry**	**Mean (SD)**
Height (m)	1.37 (0.07)
Height for age z-score	-0.22 (1.04)
	**Median (25**^**th**^**, 75**^**th**^** percentiles)**
Body mass index (kg/m^2^)	15.6 (14.6,17.3)
Body mass index for age z-score	-0.58 (-1.3, 0.37)
Body fat by bioelectrical impedance (%)	14.9 (11.5, 20.0)
Skinfold measurements (mm)	
Triceps	7.6 (5.8,11.0)
Subscapular	6.0 (4.9, 8.2)
Waist circumference, cm	57.1 (54.3, 61.0)

*SD* standard deviation

Table 2 illustrates food groupings based on their nutrient composition and culinary uses, as observed in the 24-h dietary recall. Carbohydrates from staple foods, including mixed grain porridge, “makande” (a local dish made of maize and beans), rice and stewed bananas contributed 55% of the total energy in the diet. Simple sugar contributed 12% of the diet energy, and the sources were sodas, flavoured ice pops, sweetened squeezed fruit juice, tea with sugar, and mandazi (a local deep-fried snack made of wheat and sugar). Flavoured ice pops were often reported consumed as a readily available snack within school premises. Fat contributed 16% of diet energy, and the primary sources of fats were beef, potato chips, mandazi and chapatis. Meat and alternatives reported were mostly from beef stew, fried fish, and fried chicken.

**Table 2:** Classification of foods / food groups based on 24-h recall used in dietary patterns with food frequency data

Foods from the food frequency questionnaire adapted from ISCOLE and modified based on the 24-h dietary recall (15)	Foods merged from the food frequency questionnaire (9)	Food list
Fruits	fruits	watermelon, avocado, mangoes, oranges, and ripe bananas
Vegetables	vegetables	amaranthus, spinach, cabbage, Swiss chard and pumpkin leaves, kachumbari (a salad made of onions and tomatoes)
Milk and milk products	milk and milk products	whole cow’s milk added to tea/ coffee, yoghurt, and cultured milk “mtindi” usually mixed with porridge
Chocolate and sweets	sweets and sugars	chocolates, cakes, biscuits, mandazi (a local deep-fried snack made of wheat and sugar), ice creams (industrial), flavoured ice pops “lambalamba”
Cakes and biscuits		
Doughnuts and mandazi		
Flavoured ice pops		
Commercial ice creams		
Freshly squeezed juice	sugary beverages	sodas, boxed sweetened fruit juice (commercially made), e.g., cola and sweetened squeezed fruit juice
Sweetened beverages		
Local vendor street snacks	fatty snacks	chips, samosas (deep fried snack made of minced beef, onions, spices, and wheat flour), kachori (deep fried spicy potato balls, eggs, and wheat flour) fried bananas “plantain” and cassava
Restaurants fast foods		
Beef	beef	beef
Poultry	poultry	poultry
Fish	fish	fish

Factor analysis produced two dietary patterns: a mixed dietary pattern characterized by intake of Tanzanian fatty snacks, sweets and sugars snacks, sugary beverages, meat (beef) and alternatives (fried chicken and fried fish), milk and milk products and a healthy pattern predominantly characterized by a high intake of fresh fruits and vegetables, and other foods. The mixed pattern explained 25% of the total variance in dietary intake, while the healthy pattern accounted for 15% of the total variance in dietary intake. Cumulatively, the two dietary patterns explained 40% of the total variance of the dietary intake. Factor loading for each food item is shown in Table 3. Characteristics of the study participants and anthropometric measurements across the terciles of the two dietary patterns are shown in Additional file 1.

**Table 3:** Dietary patterns created by factor analysis loadings from the food frequency questionnaire of primary school children

Variable	Factor 1 Mixed pattern	Factor 2 Healthy / fruits and vegetable pattern
Fruits	_	0.7315
Vegetables	_	0.7211
Milk and milk products	0.4599	_
Beef	0.5939	_
Chicken	0.6801	_
Fish	0.5091	_
Sugary beverages	0.5624	_
Sweets and sugars	0.4600	_
Fatty snacks	0.6551	_

Loadings based on the assigned scores from the food groups/ subgroups generated from the food frequency questionnaire; loadings with values greater or equal to 0.30 were considered and guided in the definition of the patterns. Blanks represent food groups with loading values < 0.3

Table 4 illustrates the associations between dietary patterns and adiposity outcomes. After adjusting for potential confounders (factors previously associated with overweight and obesity) for both models, we found.

**Table 4:** Adjusted estimates of the associations between dietary patterns and each adiposity (*N* = 1170)

**Variables**	**BMI z—scores**		**Body fat (%)**		**Triceps (mm)**		**Subscapular (mm)**		**Waist circumference (cm)**	
	Coef* (95% CI)	***P*-value**	Coef* (95% CI)	***P* -value**	Coef*(95% CI)	***P* -value**	Coef*(95% CI)	***P* -value**	Coef* (95% CI)	***P*-value**
**Model 1**^**a**^										
**Mixed pattern**										
Low^‡^	1.00		1.00		1.00		1.00		1.00	
Medium^‡^	1.09 (0.81,1.46)	0.6	0.96 (0.90,1.02)	0.2	0.94 (0.88,1.00)	0.1	0.97 (0.91,1.03)	0.3	1.00 (0.98,1.02)	0.9
High^‡^	1.08 (0.80,1.46)	0.6	0.96 (0.90,1.02)	0.2	0.95 (0.89,1.01)	0.1	0.94 (0.88,1.01)	0.1	0.99 (0.98,1.01)	0.4
**Healthy pattern**										
Low	1.00		1.00		1.00		1.00		1.00	
Medium	0.97 (0.71,1.32)	0.9	0.98 (0.92,1.04)	0.5	0.97 (0.91,1.03)	0.3	0.99 (0.93,1.06)	0.8	1.00 (0.99,1.02)	0.7
High	0.85 (0.63,1.15)	0.3	1.00 (0.94,1.07)	0.9	0.99 (0.93,1.06)	0.8	1.02 (0.96,1.09)	0.6	1.00 (0.98,1.02)	0.8
**Model 2**^**b**^										
**Mixed pattern**										
Low	1.00		1.00		1.00		1.00		1.00	
Medium	1.05 (0.79,1.39)	0.8	0.95 (0.90,1.01)	0.1	0.94 (0.89,1.01)	0.1	0.97 (0.91,1.03)	0.3	1.00 (0.98,1.02)	1.0
High	1.09 (0.81,1.46)	0.6	0.95 (0.90,1.01)	0.1	0.95 (0.90,1.02)	0.2	0.95 (0.89,1.01)	0.1	0.99 (0.98,1.01)	0.4
**Healthy pattern**										
Low	1.00		1.00		1.00		1.00		1.00	
Medium	0.93 (0.69,1.27)	0.7	0.98 (0.92,1.04)	0.5	0.97 (0.91,1.03)	0.3	0.99 (0.93,1.06)	0.8	1.00 (0.99,1.02)	0.6
High	0.83 (0.62,1.11)	0.2	1.00 (0.94,1.07)	1.0	0.99 (0.93,1.06)	0.8	1.02 (0.95,1.09)	0.6	1.00 (0.98,1.01)	0.7

*BMI* Body mass index

^*^Coef = coefficient

^**a**^Model adjusted for age and sex

^**b**^Model adjusted for age, sex, school type, time spent walking to school, district, availability of television and electronic gadgets at home and neighbourhood playground

^‡^ Terciles

no associations between the extracted dietary patterns and adiposity measures. There was no association between BMI z – scores categories, normal weight, thinness, and overweight/ obesity, with dietary patterns terciles.

## Discussion

This study of primary school children aged 9 – 11 years identified two major dietary patterns: a mixed pattern and a healthy pattern predominantly comprising fruits and vegetables. In this study, the prevalence of overweight and obesity was 15% (overweight 9%, obesity 6%). Since diet is not the only cause of adiposity, we controlled for other factors which might be associated with adiposity such as walking to school. We observed no significant associations between the extracted dietary patterns by factor analysis and any measure of adiposity.

From the literature, we expected an association between the intake of the mixed diet and adiposity. Although the mixed pattern in this study contained many unhealthy food items, including fatty snacks, which are linked to adiposity [32], the results did not show any associations. In contrast to these findings, reviews in children and adolescents documented that unhealthy dietary patterns, such as adherence to obesogenic diets (e.g., fast foods, sugary snacks and sweetened beverages) may result in a significant weight gain and predispose young people to overweight and obesity [20, 33]. Furthermore, other studies in children and adolescents that used factor analysis, reduced rank regression or cluster analysis to identify dietary patterns, reported a significant association between unhealthy dietary patterns and adiposity. In these studies, a westernized diet was described with frequent intake of sugary snacks and beverages, red meat, and fast foods [19, 34, 35].

Consistently with our findings, some other studies reported a lack of association between dietary patterns and adiposity. In these studies, healthy dietary pattern was characterized by frequent intake of fruits and vegetables while unhealthy pattern comprised of fast foods and sugary snacks. These studies elaborate that collapsing or grouping food items and deciding which foods could go for factor analysis was partly subjective and thus may have contributed to the lack of association [36, 37]. Another study pointed out that missing information on food portion sizes could have contributed to a lack of association [38].

A possible explanation of no association in this study could be explained by the nature of local Tanzanian snacks to which children are exposed. Local vendors within school premises usually sell local snacks such as fried cassava, chips, fried plantain, fried small fish, samosas with the local salad (“kachumbari”) and flavoured ice pops (“lambalamba”). These foods may not reflect the fast foods commonly eaten in high-income countries, and portion sizes may be too small to lead to adiposity. Further, some healthy food items, including fish, milk, and milk products, were included in the mixed pattern by factor analysis, which could have reduced associations of the pattern with adiposity or other indicators of poor health.

The strengths and limitations of this study needs to be acknowledged. We used a relatively large representative sample of school children, including a population of urban and rural primary school children, to describe the dietary patterns and their association with different adiposity measures. The use of factor analysis to study the clustered dietary behaviours and not focusing on a single food item or nutrient gives a broader picture of which foods are routinely consumed by Tanzanian school children. Further, we collected a wide range of adiposity measurements to understand their association with dietary patterns. However, the information on dietary intake was obtained through FFQ. Unfortunately, more robust quantitative methods, such as several days of a food recall or weighed food intakes, were not feasible in this study. Children needed to recall and report their food intake in a typical week which might not represent a long-term dietary habit. Thus, we cannot exclude the possibility that recall bias or lack of representativeness of the data may have affected our results. Also, our FFQ was qualitative, and therefore, we couldn’t explore portion sizes to estimate the energy intake, which could affect adiposity. Factor analysis is a data-driven approach used to identify dietary patterns existing in a particular population. However, the identification of dietary patterns is based on arbitrary decisions about naming or combining foods, which is subjective. Other types of dietary pattern analysis include cluster analysis and reduced rank regression, which are essential in examining the effects of the overall diet with diseases [39]. However, due to potential differences in nutrients among dietary patterns, and interactions with lifestyle characteristics, factor analysis may not necessarily explain the biological relationship between dietary patterns and specific health outcomes [39–41]. Finally, the study’s cross-sectional nature means we cannot infer causality and understand the contribution of dietary habits to the development of adiposity.

## Conclusion

We identified two dietary patterns for children in primary schools: mixed and healthy (fruits and vegetables). Dietary patterns were not associated with adiposity in Tanzanian primary school children, possibly because of the limitations of the FFQ, which did not record information on portion sizes. Future research should focus on understanding the essential foods / snacks consumed by school children, portion sizes and the long-term effects on adiposity in children.

## Supplementary Information

**Additional file 1.**

**Additional file 2**.

## Data Availability

All data generated and analyzed during this study are included in this article as an additional file.

## Abbreviations

FFQ: Food frequency questionnaire
BMI: Body mass index
WHO: World Health Organization
TEM: Technical Error of Measurement
ISCOLE: International Study on Childhood Obesity, Lifestyle and Environment
TFCT: Tanzania Food Composition Table
NHANES: National Health and Nutrition Examination Survey
KMO: Kaiser Meyer Olkin
