# Supplementary material for: Lack of an association between dietary patterns and adiposity among primary school children in Kilimanjaro Tanzania
Source: BMC Nutr. 2022 Apr 21;8:35. doi: 10.1186/s40795-022-00529-4 (PMC9022355; doi:10.1186/s40795-022-00529-4)
Supplement: Supplementary file 1 — Additional file 1. [file 40795_2022_529_MOESM1_ESM.docx]

|  | **Mixed pattern terciles** | | | | **Healthy pattern terciles** | | | |
| --- | --- | --- | --- | --- | --- | --- | --- | --- |
|  | **Low** | **Medium** | **High** | **P-value** | **Low** | **Medium** | **High** | **P-value** |
|  | **n (%)** | | | | | | | |
| **Age** |  |  |  |  |  |  |  |  |
| 9 | 121 (41) | 91 (31) | 86 (29) | 0.02* | 120 (40) | 88 (30) | 90 (30) | 0.01* |
| 10 | 105 (30) | 116 (33) | 129 (37) |  | 106 (30) | 134 (38) | 110 (31) |  |
| 11 | 116 (37) | 136 (36) | 125 (33) |  | 116 (31) | 120 (32) | 141 (37) |  |
| **Sex** |  |  |  |  |  |  |  |  |
| Male | 169 (37) | 142 (31) | 148 (32) | 0.09* | 173 (38) | 157 (34) | 129 (28) | 0.003* |
| Female | 173 (31) | 201 (36) | 192 (34) |  | 169 (30) | 185 (33) | 212 (37) |  |
| **Area of residence** |  |  |  |  |  |  |  |  |
| Moshi Urban | 165 (32) | 184 (36) | 163 (32) | 0.24* | 166 (32) | 163 (32) | 183 (36) | 0.20* |
| Moshi Rural | 177 (35) | 159 (31) | 177 (35) |  | 176 (34) | 179 (35) | 158 (31) |  |
| **School ownership** |  |  |  |  |  |  |  |  |
| Government | 195 (35) | 185 (33) | 183 (33) | 0.64* | 200 (36) | 193 (34) | 170 (30) | 0.06* |
| Private | 147 (32) | 158 (34) | 157 (34) |  | 142 (31) | 149 (32) | 171 (71) |  |
| **Nutritional status** |  |  |  |  |  |  |  |  |
| BMI z-score >1 (Overweight/ obese) | 60 (38) | 52 (33) | 47 (30) | 0.72* | 50 (31) | 53 (33) | 56 (35) | 0.90* |
| BMI z-score <-2 (thinness) | 33 (31) | 35 (33) | 38 (36) |  | 34 (32) | 39 (37) | 33 (31) |  |
| **Anthropometry** |  |  |  |  |  |  |  |  |
|  | **Mean (SD)** | | | | | | | |
| Height (m) | 1.37 (0.08) | 1.38 (0.74) | 1.37 (0.07) | <0.001** | 1.36 (0.08) | 1.37 (0.08) | 1.38 (0.07) | 0.003** |
| Height for age z-score | -0.27 (1.05) | -0.12 (0.99) | -0.24 (1.04) | 0.13** | -0.28 (1.06) | -0.22 (1.01) | -0.14 (1.01) | 0.20** |
|  | **Median (IQR)** | | | | | | | |
| Body mass index (kg/m2) | 15.5 (3.0) | 15.7 (3.1) | 15.6 (2.6) | 0.80^†^ | 15.6 (2.6) | 15.4 (3.0) | 15.9 (3.0) | 0.80^†^ |
| Body mass index for age z-score | -0.59 (1.76) | -0.52 (1.72) | -0.53 (1.63) | 0.90^†^ | -0.55 (1.65) | -0.69 (1.74) | -0.44 (1.68) | 0.90^†^ |
| Body fat by biological impedance (%) | 14.8 (8.3) | 15.3 (8.7) | 14.9 (8.1) | 0.59^†^ | 16.4 (7.2) | 16.4 (7.4) | 17.2 (7.4) | 0.10^†^ |
| **Skinfold measurements (mm)** |  |  |  |  |  |  |  |  |
| Triceps | 7.8 (6.2) | 7.8 (5.2) | 7.8 (4.4) | 0.90^†^ | 7.7 (4.9) | 7.0 (4.8) | 8.6 (5.6) | 0.01^†^ |
| Subscapular | 5.9 (3.8) | 6.2 (3.7) | 6.0 (2.7) | 0.40^†^ | 6.0 (2.8) | 5.7 (3.3) | 6.7 (3.9) | 0.002^†^ |
| **Waist circumference (cm)** | 57.1 (7.2) | 57.4 (7.0) | 57.0 (6.5) | 0.60^†^ | 57.0 (6.8) | 57.3 (6.9) | 57.0 (6.5) | 0.70^†^ |

Additional file 1

Baseline and anthropometric / adiposity characteristics across terciles of the two dietary patterns (mixed and healthy dietary patterns) of primary school children (N = 1170)

^*^ P-values from the Chi-square test. ** P-values from the ANOVA test. ^†^ P-values from the Kruskal Wallis test.
